# PtrWRKY19, a novel WRKY transcription factor, contributes to the regulation of pith secondary wall formation in *Populus trichocarpa*

**DOI:** 10.1038/srep18643

**Published:** 2016-01-28

**Authors:** Li Yang, Xin Zhao, Fan Yang, Di Fan, Yuanzhong Jiang, Keming Luo

**Affiliations:** 1 Key Laboratory of Eco-environments of Three Gorges Reservoir Region, Ministry of Education, Chongqing Key Laboratory of Transgenic Plant and Safety Control, Institute of Resources Botany, School of Life Sciences, Southwest University, Chongqing 400715, China; 2 Key Laboratory of Adaptation and Evolution of Plateau Biota, Northwest Institute of Plateau Biology, Chinese Academy of Sciences, 810008 Xining, China

## Abstract

WRKY proteins are one of the largest transcription factor families in higher plants and play diverse roles in various biological processes. Previous studies have shown that some WRKY members act as negative regulators of secondary cell wall formation in pith parenchyma cells. However, the regulatory mechanism of pith secondary wall formation in tree species remains largely unknown. In this study, *PtrWRKY19* encoding a homolog of *Arabidopsis WRKY12* was isolated from *Populus trichocarpa. PtrWRKY19* was expressed in all tissues tested, with highest expression in stems, especially in pith. PtrWRKY19 was located in the nucleus and functioned as a transcriptional repressor. Ectopic expression of *PtrWRKY19* in an *atwrky12* mutant successfully rescued the phenotype in pith cell walls caused by the defect of *AtWRKY12*, suggesting that PtrWRKY19 had conserved functions for homologous AtWRKY12. Overexpression of *PtrWRKY19* in poplar plants led to a significant increase in the number of pith parenchyma cells. qRT-PCR analysis showed that lignin biosynthesis-related genes were repressed in transgenic plants. In transcient reporter assays, PtrWRKY19 was identified to repress transcription from the *PtoC4H2* promoter containing the conserved W-box elements. These results indicated that PtrWRKY19 may function as a negative regulator of pith secondary wall formation in poplar.

Plant cells have a rigid cell wall that surrounds the cell membrane. In general, plant cell walls are composed of biopolymers, such as polysaccharides, phenolic compounds, and various proteins. All plant cells consist of a primary cell wall (PCW), but some specific type cells such as sclerenchyma cells also have a secondary cell wall (SCW), based on their biosynthetic composition and cellular location^1^,^2^. In stem tissues, PCW, formed at the cell plate during division, functions as a critical regulator of cell elongation and expansion^3^,^4^. In contrast, SCW, produced during the later phase of development of vascular tissues, provide mechanical strength to support physical weight of plants, and facilitate the transport of water and nutrients^5^,^6^. On the other hand, plant secondary cell walls are important for products such as paper, wood, fibers, renewable biofuel in human life^7^. Therefore, understanding the molecular mechanisms controlling SCW biosynthesis will have important implications in tree genetic improvement.

Plant secondary cell walls are mainly composed of cellulose, xylan, and lignin and their biosynthesis is regulated by a complex transcriptional network^2^,^6^. In this hierarchical network of transcription factors, SECONDARY WALL-ASSOCIATED NAC DOMAIN 1 protein (SND1/NST3) and its functional homologues (NST1 and NST2, vessel-specific VND6 and VND7) are master switches that turn on a subset of transcription factors i.e. SND3, MYB46, MYB83, MYB103^2^,^8^, which also directly activate the expression of SCW biosynthetic genes^9^. In *Arabidopsis*, AtMYB46 and AtMYB83 act as second layer-master switches of SCW biosynthesis and overexpression of *AtMYB46* and *AtMYB83* was activated the entire SCW biosynthetic pathway^9^,^10^. Other R2R3 MYB genes *PtMYB4* from *Pinus taeda*^11^, *EgMYB2* from *Eucalyptus gunnii*^12^ and *PtrMYB3* and *PtrMYB20* from *Populus trichocarpa*^13^ were also demonstrated to be functional orthologs of AtMYB46 and AtMYB83, suggesting the evolutionary conservation of the transcriptional network regulating SCW biosynthesis in vascular plants^14^.

Recent studies have shown that the NAC master switches (NST1/NST2, VND6/VND7) of SCW formation are also regulated by the upstream transcription factors. *Arabidopsis myb26* mutant exhibited a failure of anther dehiscence and male sterility^15^. A similar phenotype was observed in double mutants of *NST1* and *NST2*^16^. Overexpression of *AtMYB26* induced ectopic deposition of SCW in both *Arabidopsis* and tobacco^17^. These results indicate that MYB26 is an activator of the upstream of NAC transcription factors during SCW formation. More recently, a novel *Arabidopsis* WRKY transcription factor WRKY13 has also been shown to positively regulate lignin biosynthesis in stems by directly binding to the promoter of *NST2*^18^. In addition, some negative regulators of NAC transcription factors have been identified in plant tissues. For example, in *Arabidopsis wrky12* mutant, ectopic SCW formation appeared in pith parenchyma cells of inflorescence stems, and the expression of *NST2* activated. Further studies showed that WRKY12 protein can bind to the *NST2* promoter sequence *in vitro*, suggesting that WRKY12 negatively regulates SCW formation by directly inhibiting *NST2* expression in pith cells^19^. An *AtWRKY12* homologous gene *MIWRKY12* was also isolated from monocotyledonous grass species *Miscanthus lutarioriparius* and heterologous expression of *MlWRKY12* in an *atwrky12* background mutant successfully rescued the phenotype of pith cell walls caused by the mutation of *AtWRKY12*^20^, implying their similar functions in SCW development. However, it remains unclear whether WRKY transcription factors play also similarly critical roles in SCW development in pith cell walls of tree species.

In this study, we isolated a group IIc WRKY subfamily member *PtrWRKY19* from *P. trichocarpa.* Phylogenetic analysis revealed that PtrWRKY19 has a close relationship with MtSTP from *Medicago truncatula*^19^ and AtWRKY12. The phenotype of pith cell walls in *atwrky12* mutant could be rescued by the heterologous expression of *PtrWRKY19*. Overexpression of *PtrWRKY19* in transgenic poplar resulted in a signficant increase in pith diameter and a reduction in expression level of lignin biosynthetic genes. These results indicated that PtrWRKY19 as a function ortholog of AtWRKY12 negatively regulated SCW development in pith cells in poplar.

## Materials and Methods

### Plant materials and growth conditions

*Populus trichocarpa* plants were grown in the greenhouse at 25 °C under a 14-/10-h light/dark cycle with supplemental light (4500 lux). Seeds of the *Arabidopsis thaliana* ecotype Col-0 were incubated at 4 °C for 3 days before being surface-sterilized and germinated on 1/2 MS medium with the addition of 1.0% agar. Plants were grown in a growth chamber at 23–25 °C with the 8 h/16 h dark/light photoperiod, 70%–80% relative humidity and light intensity 150 μmol m^−2^s^−1^. The *Arabidopsis atwrky12* mutant (SALK_080995) was obtained from Arabidopsis Biological Resource Center (ABRC).

### Sequence analysis

The amino acid sequences of poplar WRKY transcription factors were obtained from website (http://www.phytozome.com). The deduced amino acid sequences were aligned with the program DNAMAN7.0 (Lynnon Corporation, USA). The phylogenetic relationships of WRKY proteins were analyzed with the neighbour-joining method using MAGE 5.0^21^. The localization of the PtrWRKY19 protein was predicted by the WoLF PSORT program (http://wolfpsort.org/)^22^.

The GenBank accession numbers of the WRKY genes from different species were: MlWRKY12 (KC191597), MtSTP (HM622067), AtWRKY12 (At2g44745), OsWRKY36 (BK005039), AtWRKY13 (AF421153), VvWRKY20 (JQ782602), AtWRKY25 (AT2G30250), OsWRKY24 (AY676925), PtrWRKY13 (POPTR_0005s08860.1), PtrWRKY19 (POPTR_0014s04890.1), PtrWRKY25 (POPTR_0007s06930.1), OsWRKY78 (BK005212), VvWRKY2 (AY596466), PtrWRKY3 (POPTR_0008s09140.1), PtrWRKY4 (POPTR_0017s12430.1), PtrWRKY68 (POPTR_0004s12000.1), AtWRKY3 (At2g03340), AtWRKY4 (AF425835).

### Gene expression analysis

Poplar tissues were collected from 6-month-old stems, flash frozen in liquid nitrogen and stored in a −80 °C freezer. Total RNA was isolated from different tissues of poplar plants according to Zhong *et al.*^14^. The 2^nd^ and 6^th^ leaves from the apex of plants were defined as young and mature leaves, respectively. Different tissues of stems were collected from the 2^nd^ to 7^th^ internodes. The pith/xylem tissues were separated using microknives under a stereoscopic microscope. First-stand cDNAs were treated with DNase I (Promega, USA) and then used for quantitative real-time PCR (qRT-PCR) analysis. Real-time PCR was performed on a TP700 real-time PCR machine (TaKaRa, Japan) using the SYBR Green master mix reagent (TaKaRa, Dalian, China). *Ptr18S* rRNA was used as the reference gene for internal standardization of real-time PCR data. The PCR primers used for qRT-PCR were listed in Supplementary Table S1.

### Subcellular localization

The full-length of *PtrWRKY19* was fused in frame with *GFP* cDNA and ligated into pCX-DG^23^. The *35S-PtrWRKY:GFP* fusion construct was introduced into onion epidermal cells by particle bombardment (GJ-1000, SCIENTZ, China). GFP fluorescent images were examined with a confocal microscope (Olympus FV500) at 18 h after bombardment. The onion skin was stained with DAPI, and then photographed by the confocal microscopy (Leica TCS SP5).

### Transcriptional repression in yeast

To determine the transcription activity of PtrWRKY19, the GAL4BD/UAS/LacZ transcient assays were performed in yeast cells. Yeast GaL4BD expression vectors were obtained from Clontech. The cDNAz encoding PtrWRKY19 was amplified by PCR and cloned into *Eco*RI and *Bam*HI sites of pGBKT7 vector. Yeas strains harboring *UAS-LacZ* reporter construct were grown to late logarithmic phase, harvest by centrifugation and resuspended in 200 μl of breaking buffer containing 100 mM potassium phosphate, 0.5 mM dithiothreitol, protease inhibitor mixture, 0.2% Triton X-100, pH 7.8. The β-galactosidase activity was determined at least three independent clones using detection kit Galacto-Light (Tropix, Bedford, MA).

### Gene cloning and vector construction

The cDNA fragment encoding *PtrWRKY19* was amplified in PCR reaction mixture of 50 μl, which contained 2.5 μl cDNA, 10 × PCR buffer, 0.3 mM dNTP, 2 units of pfu DNA polymerase (Takara, Dalian, China) and 0.5 μM of each primer. The reaction program consisted of 34 cycles of 94 °C for 45 s, 54 °C for 45 s and 72 °C for 90 s, followed by a final extension of 72 °C for 10 min. The PCR product was purified and cloned into the plant binary vector pCXSN^23^ and transformed into *E. coli* strain DH5á and sequenced by BGI (Beijing, China). Primers used for gene cloning are provided in Supplementary Table S1.

Genomic DNA was isolated from mature leaves of poplar plants using cetyl trimethylammonium bromide method. According to the sequences deposited in the *Populus* genome database, an about 1.5 kb of upstream sequence of *PtrWRKY19* was cloned and inserted into plant binary vector pCXGUS-P^23^. The PCR fragments were verified by DNA sequencing analysis by BGI (Beijing, China) in both directions.

These plant binary constructs were transformed into *Agrobacterium tumefaciens* EHA105 by the freeze-thaw method^24^.

### Transformation of *Arabidopsis* and *P. tomentosa* Carr

*A. tumefaciens* strain EHA105 containing the *PtrWRKY19*:GUS promoter construct was used to transform *A. thaliana* (Col-0) plants *via* the floral dip method^25^. Transgenic lines were selected on MS media containing 40 mg.l^−1^ hygromycin and grown in a growth chamber under long-day conditions (23–25 °C with the 8 h/16 h dark/light photoperiod).

Transgenic poplar plants were generated by *Agrobacterium*-mediated transformation as described previously^26^. Recombinant *Agrobacterium* cells were used to infect poplar leaf discs and putative transgenic plants were selected on woody plant medium (WPM)^27^ supplemented with 9 mg l^−1^ hygromycin. Rooted plantlets were acclimatized in pots at 25 °C °C in a 14/10 h light/dark cycle and then transferred to the greenhouse for further studies.

### Transient expression in tobacco leaves

The promoter fragment of the poplar *C4H2* gene was amplified by PCR with the gene-specific primers described in Supplementary Table S1. These fragments were individually fused to the *GUS* reporter gene in the pCXGUS-P vector to generate reporter constructs. *Agrobacterium* cells carrying *35S-PtrWRKY19* construct were used as an effector. Co-expression experiments were performed according to the method described by Zhong *et al.*^14^. *Agrobacterium* strains EHA105 containing the *PtrWRKY19*-*GUS* or *35S*-*PtrWRKY19* construct, were co-infiltrated in fully expanded leaves of tobacco plants (*Nicotiana benthamiana*) using a 1 ml syringe. After agroinfiltration, plants were covered with a transparent plastic cover and transferred into a growth chamber at 25 °C with 16/8 h light/dark cycle for 2–3 days. Quantitative GUS assays were carried out on the infiltrated zone using 4-methylumbelliferyl-b-D-glucuronide as substrate^28^. GUS activities were estimated as the mean of three independent assays.

### Histochemical staining for GUS activity

Histochemical staining of GUS activity was performed according to the method described previously^29^. Tissues of transgenic poplar plants were immersed into GUS reaction buffer [1 mM X-Gluc (5-bromo-4-chloro-3-indolyl-β- d-glucuronide), 100 mM phosphate buffer pH 7.0, 0.1% Triton X-100, 5 mM K_3_Fe(CN)_6_, 5 mM K_4_Fe(CN)_6_, 10 mM EDTA and 20% methanol]. After 2–4 h incubation in dark at 37 °C, stained samples were bleached with absolute ethyl alcohol: acetic acid =7:3 (v/v) and then photographed by light microscopy (Olympus ZX16).

### Tissue sections and microscopy analysis

Inflorescence stem samples of *Arabidopsis* and the 5^th^ internode on the stems of 6-month-old poplar plants were fixed in FAA buffer (formaldehyde:glacialacetic acid:50% ethanol, 1:1:18), and then embedded in paraffin. Specimens were cut into 8-μm-thick sections (Thermo Finesse 325 rotary microtome) and stained with 0.05% (w/v) toluidine blue O or phloroglucinol-HCl and observed under Zeiss Axio Scope (Zeiss, Oberkochen, Germany).

## Results

### Isolation and characterization of PtrWRKY19

**Figure 1:**
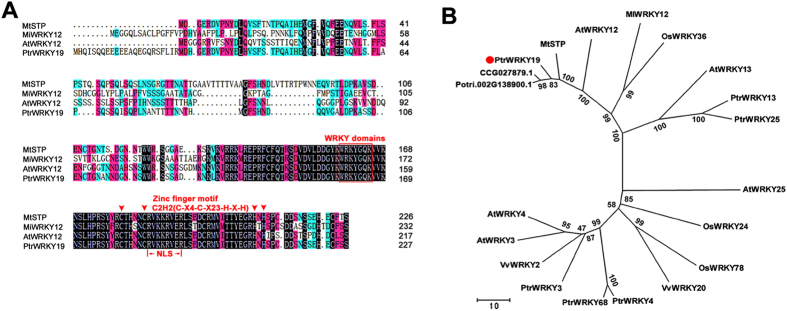
Phylogenetic relationship of WRKY proteins. (**A**) Multiple sequence alignment of PtrWRKY19 and other WRKY domain proteins. Identical amino acids are shaded in gray. The conserved WRKY domain and C2H2 zinc finger motif are marked by a line and triangles, respectively. (**B**) Phylogenetic tree of the deduced amino acid sequences of PtrWRKY19 and other WRKY proteins from *Arabidopsis*, rice, *Medicago* and grape. Phylogenetic analysis was performed by the neighbour-joining (NJ) method using MEGA version 5.0.4 Scale bar corresponds to 10 estimated amino acid substitutions per site.

To isolate the orthologous gene of *Arabidopsis AtWRKY12* from *Populus trichocarpa,* we used the AtWRKY12 amino acid sequence as a query sequence to blast in the *P. trichocarpa* genome database (http://www.phytozome.com/). A putative WRKY transcription factor, named PtrWRKY19 according to its structural feature^30^ was assembled in a contig and the full-length cDNA was amplified from total poplar leaf cDNA using the specific oligo-nucleotide primers (Supplementary Table S1). Sequence analysis showed that the cDNA sequence of PtrWRKY19 encodes a protein of 228 amino acid residues (Fig. 1A) with a predicted molecular mass of 26.4 kD and a calculated pI of 6.55. The deduced amino acid sequence of PtrWRKY19 contains a conserved WRKY domain at the C-terminal end along with a C_2_-H_2_ (C-X_4_-C-X_23_-H-X-H) type zinc-finger-like motif (Fig. 1A), which is the unique zinc ligands among WRKY domain belonging to group I and II^31^. In addition, a putative nuclear localization signal (NLS) was found in the C-terminal region of PtrWRKY19 protein (Fig. 1A).

A phylogenetic tree was constructed using the neighbor-joining method with the amino acid sequences of PtrWRKY19 and other plant WRKY proteins (Fig. 1B). PtrWRKY19 shared high similarity to CCG027879.1 (*P. euphratica*, 97.8%), Potri.002G138900.1 (*P. trichocarpa*, 89.8%), MtSTP (*Medicag truncatula*, 74.6%) and AtWRKY12 (*Arabidopsis*, 61.5%). Based on the classification methods reported previously^30^,^31^, PtrWRKY19 belongs to group IIc family (Fig. 1B), members of which contain only one WRKY domain and a C_2_-H_2_ type zinc-finger-like motif in the C-terminal.

### Expression patterns of *PtrWRKY19*

**Figure 2:**
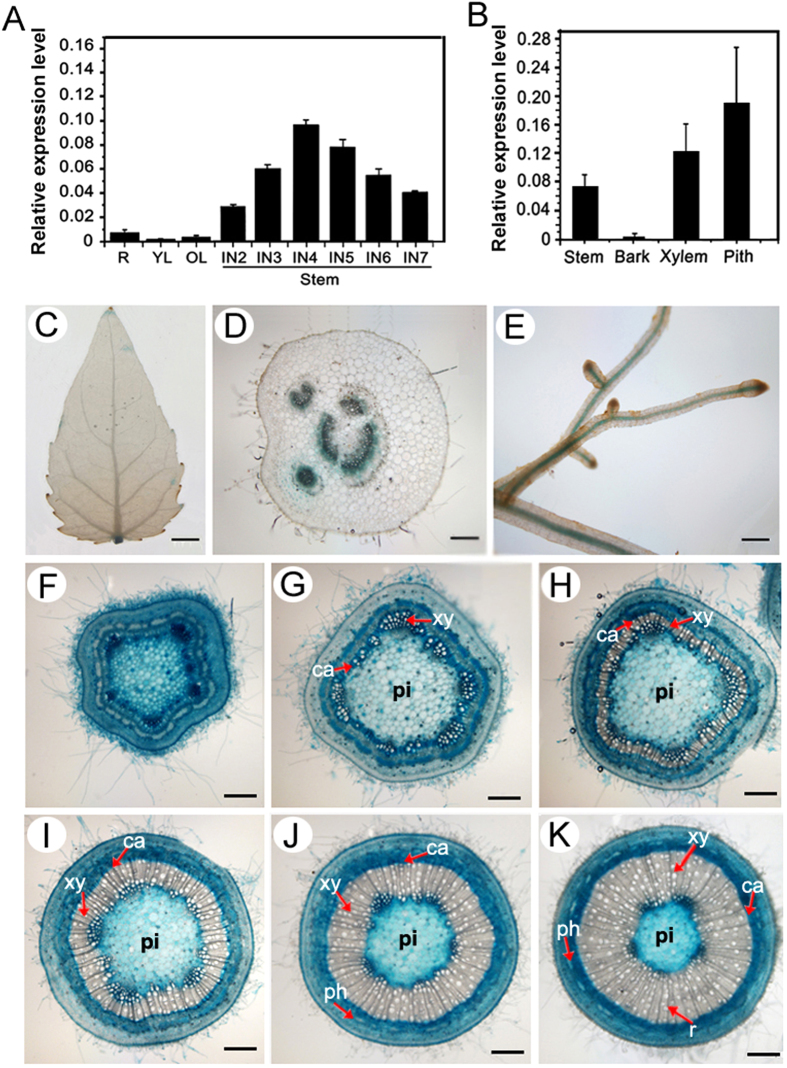
Expression pattern of *PtrWRKY19* in poplar. Quantitative real-time (qRT) PCR analysis of *PtrWRKY19* transcript levels in various organs (IN, internode) of poplar. R: roots; YL: young leaves (the second leaf); OL: old leaves (the sixth leaf from the apex). (**B**) qRT-PCR analysis of *PtrWRKY19* expression in different tissues of the stems at the 6^th^ internode. Poplar *18S* was used as an internal control. Bars represent ± SD. The *PtrWRKY19* gene promoter-driven GUS expression vector was engendered and introduced into *P. tomentosa* Carr. Gus staining pattern of 4-week-old transgenic plants: (**C**) leaves; (**D**) petioles; (**E**) roots; (**F–K**) transverse sections of the stem at the different internodes (from 2^nd^ to 7^th^ internode). Scale bar: C = 0.5 cm; D, E = 2.5 mm; F-K = 200 μm. Cambial zone (Ca), Phloem (ph), Pith (pi), Ray (r), Xylem (Xy).

To determine the expression of PtrWRKY19 in different tissues of poplar plants, total RNA was isolated and mRNA was reversely transcribed into cDNA as a template for a real-time RT-PCR analysis. *PtrWRKY19* was expressed in all the tissues examined, with highest expression in stems (Fig. 2A), especially in pith tissues (Fig. 2B). During internode development, *PtrWRKY19* transcript level increased gradually with maturity and highest expression was found in the fourth internode (Fig. 2A).

In order to further investigate the tissue-specific expression pattern of *PtrWRKY19*, a 1.5 kb promoter fragment of *PtrWRKY19* was fused to a *GUS* reporter gene, and the recombinant gene was introduced into *P. tomentosa* Carr. Histochemical GUS staining showed weak GUS activity in leaves (Fig. 2C) and strong staining reactions in all vascular tissues of petioles and roots (Fig. 2D,E). In young stem tissues (2^nd^ and 3^rd^ internodes), strong GUS activity was observed in all tissues including sclerenchyma and parenchyma cells (Fig. 2F,G). In older stem internodes (from 4^th^ to 7^th^), a strong GUS expression was detected in the phloem, cambium, developing xylem and stem pith cells, whereas mature xylem showed a faint GUS staining (Fig. 2H–K). In addition, the *GUS* gene under the control of the *PtrWRKY19* promoter was transformed into *A. thaliana*. GUS activity was detected in all vascular tissues of transgenic plants, including veins, roots, tricomes, inflorescence stems and flowers (Supplementary Figure S1). These results indicate that PtrWRKY19 might be involved in SCW formation in stem tissues, especially in pith parenchyma cells.

### PtrWRKY19 is localized in the nucleus and functions as a transcriptional repressor

**Figure 3:**
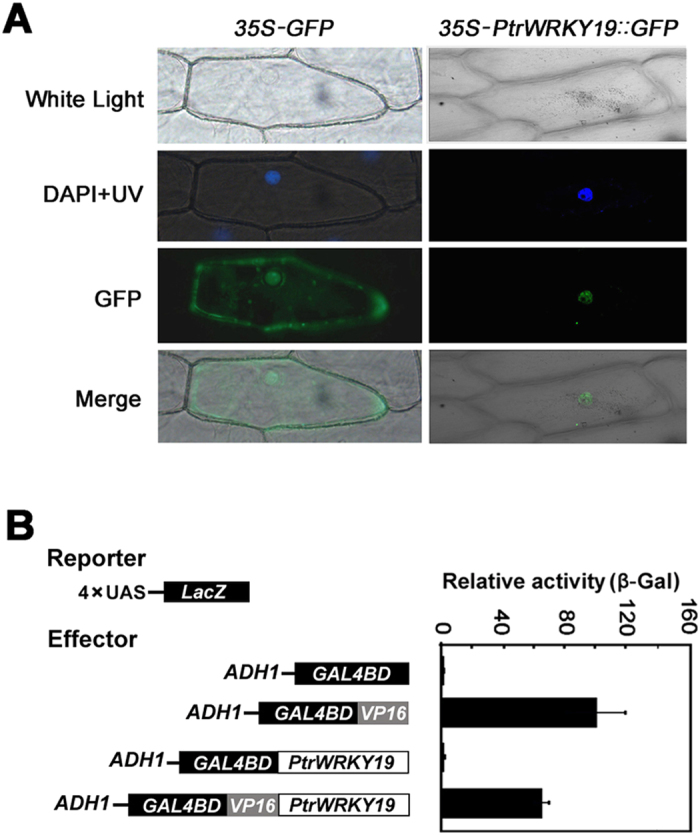
Subcellular localization and transactivation assays of PtrWRKY19. Onion epidermis was transformed with *35S-PtrWRKY19::GFP* and *35S-GFP* constructs by particle bombardment. The position of nucleus was ensured by DAPI staining and bright-field images were compared. In this experiment, *35S-GFP* was used as control. (**B**) Transcriptional activation analysis of PtrWRKY19 analyzed by the chimeric reporter/effector assay in yeast on the plates with solid SD medium. Data represent mean of three biological repeats ± SD. GAL4DB null vector was used as a negative control and GAL4DB fused with VP16 was used a positive control.

The nuclear localization signal (NLS) prediction by WoLF PSORT software (http://wolfpsort.org/) showed that PtrWRKY19 contains a NLS sequence (RVKKRVER)^32^ (Fig. 1A). To determine whether PtrWRKY19 functioned as a transcription factor, we first analyzed its subcellular localization. PtrWRKY19 was fused to green fluorescent protein (GFP) under the control of the cauliflower mosaic virus (CaMV) 35S promoter. The fusion construct of *35S-PtrWRKY19*::*GFP* and the control vector *35S-GFP* were introduced into onion epidermal cells by particle bombardment. The green fluorescence of PtrWRKY19::GFP recombinant protein was exclusively detected in the nucleus of onion cells (Fig. 3A), suggesting that PtrWRKY19 localizes in the nucleus. In contrast, the *35S-GFP* protein occurred throughout the cells including cytoplasm and the nucleus (Fig. 3A).

To evaluate the transcriptional activity of PtrWRKY19 protein, the complete PtrWRKY19 was fused with GAL4:VP16 (*Herpes simplex virus* activation domain) protein. The activity of PtrWRKY19 as a transcription factor was determined by co-transfection of effector and various reporter constructs into yeast cells and measurement of reporter enzyme activity (Fig. 3B). The relative activity of reporter β-galactosidase in the existence of GAL4DB:VP16:PtrWRKY19 was distinctly reduced compared with that of control (the presence of GAL4DB:VP16 effector) (Fig. 3B), indicating that PtrWRKY19 is a transcriptional repressor.

### PtrWRKY19 can complement the Arabidopsis wrky12 mutant

**Figure 4:**
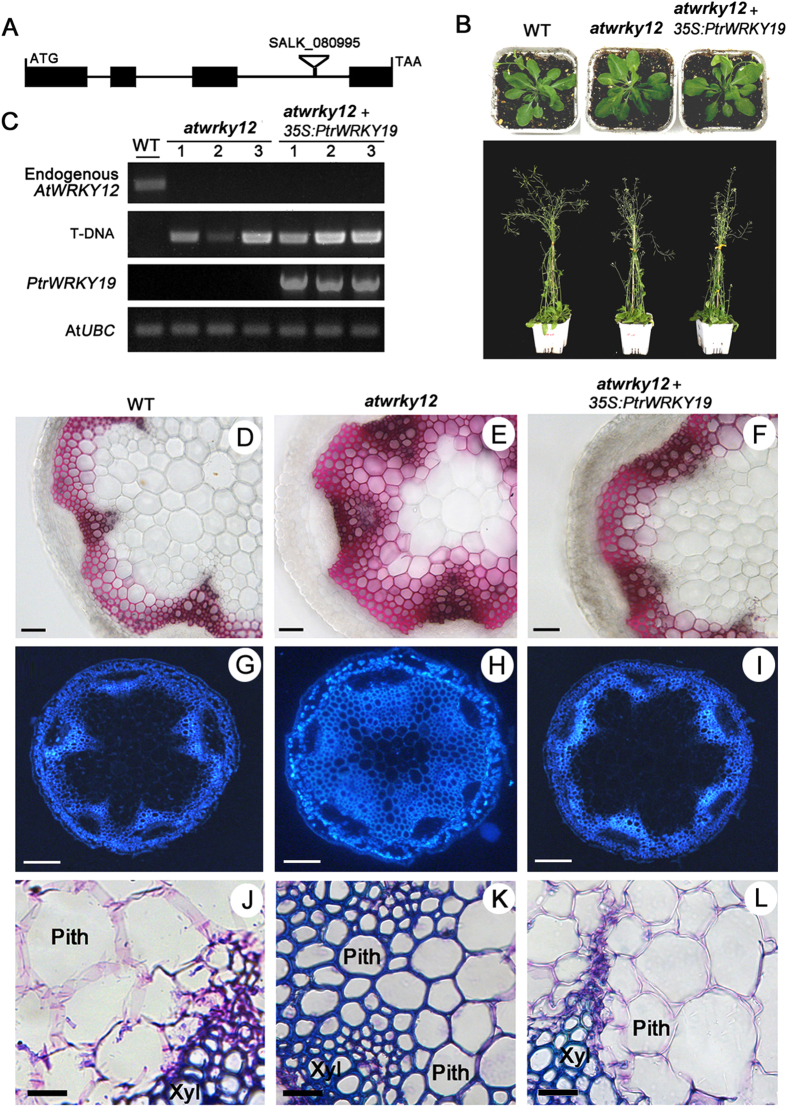
Complementation of the *Arabidopsis wrky12* mutant with *PtrWRKY19*. (**A**) *AtWRKY12* gene structure and T-DNA insertion site. (**B**) Visible phenotypes of WT, *wrky12* mutant and *wrky12* mutant transformed with *35S-PtrWRKY19* gene. (**C**) Expression levels of *AtWRKY12* and *PtrWRKY19* in WT, *wrky12* mutant and transgenic plants by PCR analysis. *AtUBC* gene was used as the control. (**D–F**) Phloroglucinol staining of the stems from WT, *wrky12* mutant and *wrky12* mutant complemented with *35S-PtrWRKY19* gene, respectively. (**G–I**) UV autofluorescence of cross-sections of the stems from WT, *wrky12* mutant and *wrky12* mutant complemented with *35S-PtrWRKY19* gene, respectively. (**J–L**) Light microscopy of pith cell walls by toluidine bule staining. Xyl, Xylem. Scale bar represent 50 μm (**D–F**); 100 μm (**G–I**); 20 μm (**J–L**).

A previous study has demonstrated that loss-of-function of *AtWRKY12* in *Arabidopsis* or its ortholog in *M. truncatula* results in SCW thickening in pith cells involved in ectopic deposition of lignin, xylan, and cellulose^19^. To confirm whether PtrWRKY19 is a functional ortholog of AtWRKY12 in poplar, the *PtrWRKY19* gene under the control of the *CaMV 35S* promoter was transformed into homozygous *AtWRKY12* mutant plants (SALK_080995), *atwrky12* (Fig. 4A). More than twenty independent transgenic lines were obtained. Most of them showed a phenotype that resembled wild-type plants (Fig. 4B) and only a few (3 lines) displayed retarded growth (data not shown). RT-PCR analysis further confirmed the expression of *PtrWRKY19* in *atwrky12* mutant and transgenic plants (Fig. 4C). Determination of dry weights showed that a significant increase in biomass density was found in *atwrky12* mutant lines compared to the wild-type (WT), whereas no obvious reduction did seem to occur in transgenic plants overexpressing *PtrWRKY19* (Supplementary Figure S2).

Moreover, stem sections of WT, *atwrky12*, and *atwrky12*+*35S-PtrWRKY19* were treated with phloroglucinol-HCl. The red color showed that ectopic composition of lignin was detected in the pith parenchyma cells of the *atwrky12* mutant (Fig. 4E). In contrast, no staining signal was found in the pith cells of WT and *atwrky12*+*35S-PtrWRKY19* plants (Fig. 4D,F). The lignin pattern of stem sections was visualized by UV autofluorescence. Strong signals for lignin were observed in the xylem and pith cells of the *atwrky12* mutant (Fig. 4H), whereas lignin autofluorescence was only detected in the xylem cells of WT and *atwrky12*+*35S-PtrWRKY19* lines (Fig. 4G,I). In addition, toluidine blue staining also showed that the lignified pith cell walls in the mutant were obviously thicker than in the WT and *atwrky12*+*35S-PtrWRKY19* plants (Fig. 4J–L). These results showed that PtrWRKY19 is an ortholog of AtWRKY12 in poplar and functions as a negative regulator of SCW development in pith cells.

**Figure 5:**
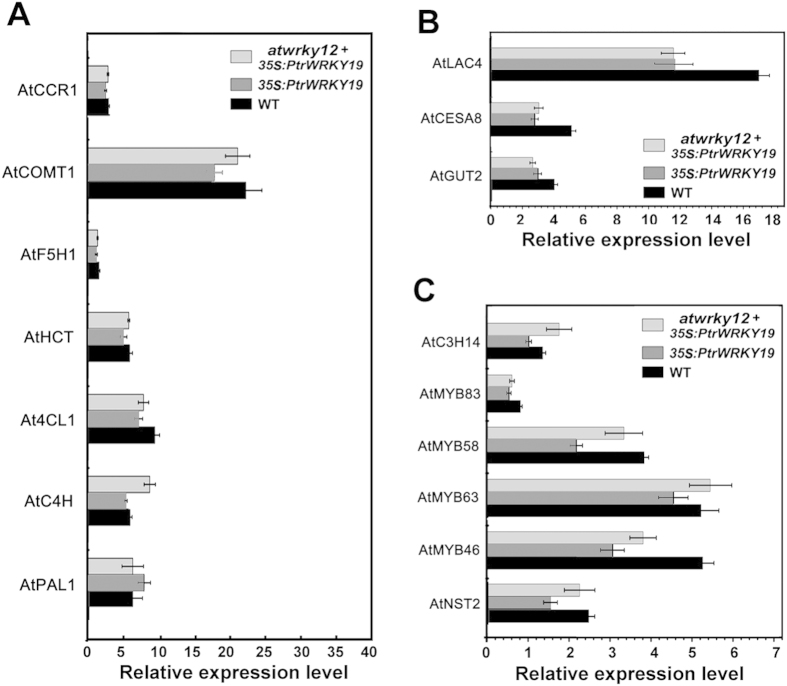
Transcript levels of SCW biosynthesis-related genes in WT, *35S-PtrWRKY19* and *wrky12* mutant complemented with *35S-PtrWRKY19* gene. Transcript levels of structural genes in lignin biosynthesis pathway. COMT, Caffeic acid O-methyltransferase; PAL, phenylalanine ammonialyase; HCT, hydroxycinnamoyl transferase; F3H, Flavanone 3-hydroxylase. HCT, p-hydro-xycinnamoyl-CoA; C4H, cinnamate 4-hydroxylase; CCR, cinnamoyl-CoA reductase; 4CL, 4-coumarate:CoA-ligase; CAD, cinnamyl alcohol dehydrogenase. (**B**) Expression levels of cellulose, xylan biosynthetic genes. LAC, Laccase; CesA, cellulose synthase; GUT, glucuronosyl transferase. (**C**) Transcript levels of SCW regulatory genes. The *AtUBC* gene was used as the internal reference. Data points shown are means from three biological replicates (individual lines). Each bar indicates ± SD of triplicate experiments.

To elucidate the transcriptional regulatory mechanism of PtrWRKY19 underlying the control of SCW development of pith cells, the expression levels of genes involved in SCW biosynthesis in WT, *35S-PtrWRKY19* and *atwrky12*+*35S-PtrWRKY19* plants were determined. As shown in Fig. 5A,B, overexpression of *PtrWRKY19* in wild-type and *atwrky12* background did not seem to repress the expression of lignin biosysnthetic genes (*AtCCR1*, *AtCOMT1*, *AtF5H1*, *AtHCT*, *At4CL1*, *AtC4H*), but down-reglated the expression of xylan biosynthetic gene (*AtGUT2*), laccase gene (*AtLAC4*) and cellulose synthase gene (*AtCES8*), compared to the wild-type plants. To investigate whether overexpression of *PtrWRKY19* regulated expression of the transcription factor genes involved in SCW formation, transcript levels of some NAC and MYB genes in transgenic plants were tested by qRT-PCR. The results revealed that expression of these transcriptional regulators was repressed in *35S-PtrWRKY19* plants compared to the wild-type control (Fig. 5C). But in *atwrky12* background, overexpression of *PtrWRKY19* did not result in decreased expression of these genes.

### Overexpression of *PtrWRKY19* in *P. tomentosa* Carr

**Figure 6:**
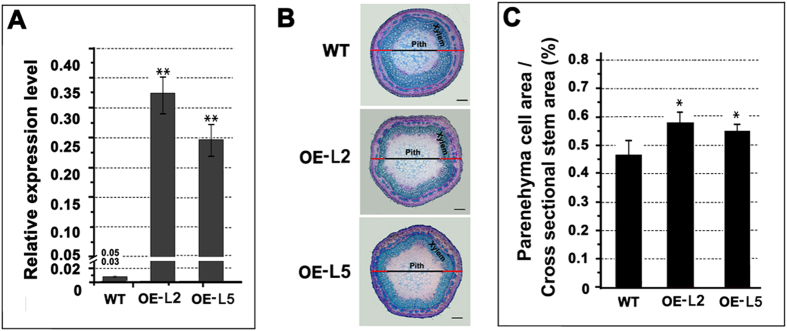
Effect of *PtrWRKY19* overexpression in transgenic poplars. (**A**) Quantitative RT-PCR analysis of *PtrWRKY19* in the wild type and two independent *35S-PtrWRKY19* lines. Poplar *18S* was used as an internal control. (**B**) Transverse sections of the stems from the wild type and transgenic plants. (**C**) Parenehyma cell area measurements of the stems. Values are means ±SD for n = 4. Asterisks above the bars indicate values determined by Student’s *t* test to be significantly different from control (**P < 0.01).

To further investigate whether PtrWRKY19 is involved in the regulation of SCW development in pith cells of poplar, the *35S-PtrWRKY19* fusion gene was introduced into Chinese white poplar (*P. tomentosa* Carr.) *A. tumefaciens*-mediated transformation. More than ten hygromycin-resistant putative transformants were obtained and PCR analysis was used to confirm the integration of the transgenes in transformed plants (Supplementary Figure S3). Expression of *PtrWRKY19* in transgenic plants were determined by qRT-PCR and these lines (L2 and L5) with significantly high transcript level of *PtrWRKY19* were chosen for further analysis (Fig. 6A). No phenotypic changes were observed in all generated transgenic plants compared with the wild type (Supplementary Figure S4). Histological analyses of xylem were performed using the natural autofluorescence of phenolic compounds under UV-light. Less lignin autofluorescence was detected in cross-sections of *35S-PtrWRKY19* lines as compared with that of the control (Supplementary Figure S5). Toluidine blue staining showed that although stem cross-sectional area of transgenic *35S-PtrWRKY19* plants was similar to that of the wild type, the cross-sectional area of parenchyma cells in pith significantly increase in transgenic *35S-PtrWRKY19* plants (Fig. 6B,C), indicating that secondary wall thickening was repressed in xylem of transgenic plants overexpressing PtrWRKY19 compared to wild-type plants.

**Figure 7:**
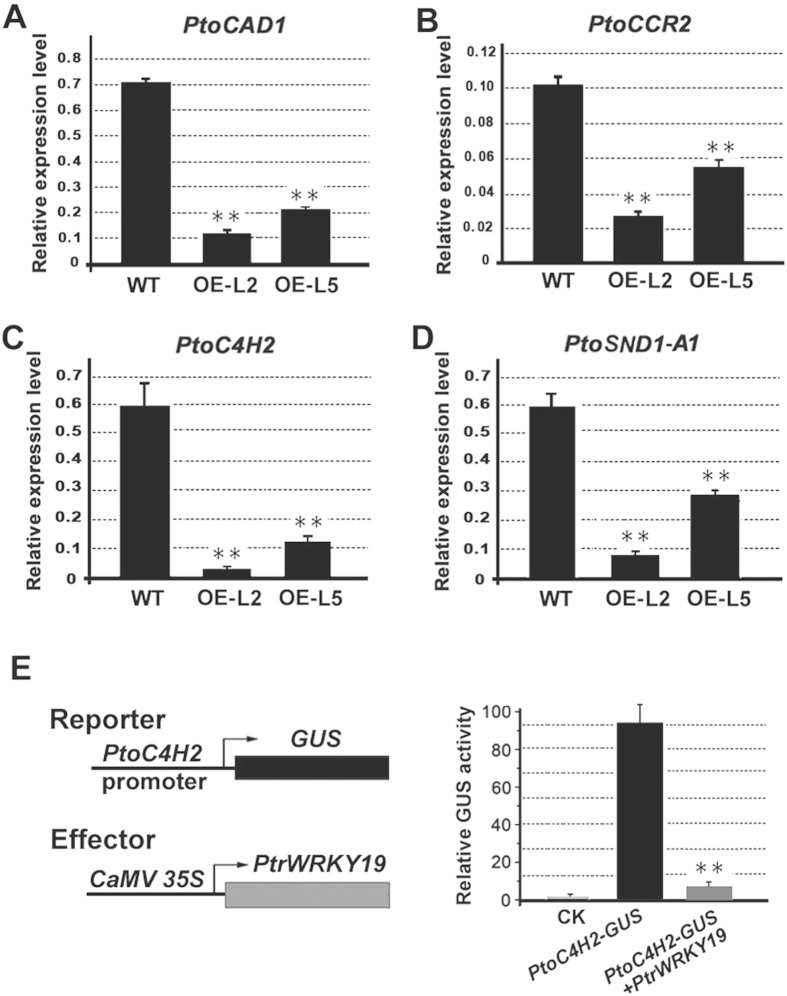
PtrWRKY19 protein represses the expression of lignin pathway genes and SCW-related genes in transgenic plants. (**A–D**) Quantitative RT-PCR was used to analysis expression levels of *PtoCAD1*, *PtoCCR2*, *PtoC4H2* and *PtoSND1-A1*, respectively. (**E**) PtrWRKY19 binds to the *PtoC4H2* promoter. Schematic of the *PtoC4H2*-GUS reporter (top) and *PtrWRKY19::GFP* effectors (bottom). Transcription level of the *PtoC4H2* promoter reporter by PtrWRKY19 was assessed by GUS activity. *PtoC4H2-*GFP was used as a control. Error bars represent ±SD from 3 independent experiments. **P < 0.01, Student’s *t* test.

To test the prediction that PtrWRKY19 is involved in pith SCW formation in poplar, the expression of lignin biosynthetic genes in transgenic *35S-PtrWRKY19* plants was examined by qRT-PCR. Reduced transcript levels were observed for the *PtoCAD1*, *PtoCCR2* and *PtoC4H2* genes (Fig. 7A–C), suggesting an effect on the lignin biosynthesis genes. In previous reports, WRKY12 and its orthologs negatively regulate the expression of *NST2*, which acts as master regulators play a critical role in switching on and off secondary wall biosynthesis^33^, and represses cell wall biosynthetic genes in pith cells^19^,^20^. We also determined the level of *PtoSND1-A1* encoding NAC secondary wall thickening pro-moting factor in poplar and found that *PtrWRKY19* overexpression reduced significantly (*P* < 0.01) transcript level of *PtoSND1-A1* (Fig. 7D).

Previous studies showed that WRKY transcription factors specifically bind to W-box elements in their target gene promoters^34^. To determine the relationship between PtrWRKY19 and its target gene promoters, the *PtoC4H2* promoter with at least two predicted W-box elements (Supplementary Figure S6) were transiently expression in tobacco leaves using a chimeric reporter/effector assay. In this assay, the effector construct containing *PtrWRKY19* driven by the CaMV 35S promoter was cotransformed with the reporter construct harboring the β-glucuronidase (GUS) reporter gene driven by the full-length *PtoC4H2* promoter (Fig. 7E). Compared with the GUS activity in tobacco cells carrying the *PtoC4H2-GUS* gene, co-expression of the *35S-PtrWRKY19* and *PtoC4H2-GUS* gene resulted in significantly reduced (*P *< 0.01) by approximately 12-fold (Fig. 7E), suggesting that PtrWRKY19 acts as a negative regulator of SCW formation in pith cells in poplar.

## Discussion

In vascular plant, stems provide mechanical strength to support the plant’s weight, and conduct the transport of water, hormones and minerals. The stem has three simple cell types: the parenchyma, collenchyma, and sclerenchymacells. Parenchyma cells have thin primary walls and occur in pith, cortex and epidermis. In contrast, sclerenchyma cells, such as xylem and phloem, have thick lignified secondary walls^35^. In dicotyledonous plants, the pith parenchyma cells, which are encircled by a ring of xylem with secondary walls, have only primary cell walls, indicating that there exists a conserved mechanism for inhibiting secondary growth in pith cells. In this study, we have identified a transcriptional regulator PtrWRKY19 from poplar, the amino acid sequence of which shows homology to the conserved WRKY domain of various WRKY transcription factors in responsible for the parenchymatous nature of the pith cells (Fig. 1A). The heterologous expression of PtrWRKY19 in *atwrky12* mutant plants successfully complemented the phenotype caused by the loss-of-function of *AtWRKY12* (Fig. 4), which is a negative regulator of SCW formation in pith parenchyma^19^. Therefore, these results suggested that PtrWRKY19 is involved in negatively regulating secondary wall development in pith tissues of poplar.

The WRKY family proteins are well known for their involvement in diverse physiological and developmental processes, especially in the regulation of plant stress tolerance^31^,^36^,^37^. Recently, it has been demonstrated that WRKY transcription factors also play important regulatory roles in regulating the biosynthesis of many phenolic compounds, such as flavonoids^38^. For example, the *Arabidopsis* WRKY transcription factor TTG2 has been reported to be involved in condensed tannins and mucilage production in the seed coat, in a TTG1-dependent way^39^. A novel function of the WRKY transcription factors is to inhibit pith secondary wall formation^19^,^20^. The *Arabidopsis* AtWRKY12 gene was predominantly expressed in pith cells and *atwrky12* loss-of-function mutants showed induced secondary wall formation of pith cells^19^. Similarly, in monocotyledonous species *M. lutarioriparius*, *MlWRKY12* was expressed in vascular bundle sheath, sclerenchyma and parenchyma tissues and heterologous expression experiments showed that MlWRKY12 can complement *Arabidopsis atwrky12* mutant^20^. In grapevine, *VvWRKY2* was specifically expressed in cells undergoing ligification in young stems. Transgenic tobacco plants over-expressing *VvWRKY2* exhibited altered expression of genes involved in lignin biosynthesis pathway and cell wall formation^40^. In this present reports, poplar PtrWRKY19 exhibited typical features of WRKY transcription factors (Fig. 1A) and was highly expressed in stems, especially in pith cells (Fig. 2B). GUS activity analysis revealed that *PtrWRKY19* was also expressed in vascular tissues of roots and petioles (Fig. 2D,E), inconsistent with that of *AtWRKY12* which specifically expressed in pith and cortex cells of stems but not in roots^19^. When *PtrWRKY19* was constitutively expressed in *Arabidopsis* and *wrky12* mutant plants, transgenic plants exhibited normal phenotypes compared with the wild type (Fig. 4B). Quantitative RT-PCR analysis indicated that s few SCW biosynthetic genes were down-regulated in transgenic plants overexpressing *PtrWRKY19*, suggesting that PtrWRKY19 is a negative factor for SCW formation in pith cells.

WRKY proteins can act as transcriptional activators or repressors^41^. Among the 4 characterized WRKY proteins involved in regulating SCW formation in pith cells, *Arabidopsis* AtWRKY12, *Miscanthus* MlWRKY12 and *M. truncatula* MtSTP are transcriptional repressors^19^,^20^, whereas only AtWRKY13 shows transactivation activity^42^. Interestingly, all of these proteins belong to group II WRKYs, which contain one WRKY motif and one C2H2-type zinc-finger motif (Fig. 1). PtrWRKY19, an ortholog of AtWRKY12, had similar function in SCW formation in pith cells in poplar. *In vivo* assay showed that PtrWRKY19 had transcriptional repressor activity in yeast cells (Fig. 3). In addition, a putative WRKY transcription factor Potri.002G138900.1, which shares high similarity to PtrWRKY19 (Fig. 1B), was found in the poplar genome. However, it remains unclear whether there exist other homologs of PtrWRKY19 in the genome.

Almost all WRKY transcription factors bind preferentially to W-box elements containing a “TGAC” core sequence in their target gene promoters^31^. In *Arabidopsis*, direct binding of *AtWRKY12* to the *NST2* promoter and repression of *NST2* expression by WRKY12 were confirmed by EMSA and in *planta* transgenic experiments^19^. Similar results were obtained in transgenic *35S:AtWRKY13* plants by performed *in vivo* chromatin immunoprecipitation (ChIP) assays^18^. In transgenic poplar overexpressing *PtrWRKY19*, the expression levels of lignin biosynthetic genes and NAC transcription factor were reduced (Fig. 7A–D). Transient expression assays showed that PtoC4H2 with several W-box elements was s downstream target of PtrWRKY19 and its expression was repressed by PtrWRKY19 (Fig. 7E). These results indicated that there exists a conserved mechanism in regulating SCW formation in pith cells in herbaceous and woody plants.

In *A. thaliana* and *M. truncatula*, loss of WRKY expression in mutants resulted in derepression of NAC and MYB46 transcription factors and this activation turned on SCW formation in pith cells in which parenchyma cells normally have only thin primary walls^19^. The secondary thickening of pith cells in *Araibdopsis wrky12* mutant results in an approximately 50% increase in biomass density in stem tissue^19^. In this study, interestingly, secondary wall thickening in xylem of transgenic poplar plants overexpressing *PtrWRKY19* appeared to be repressed and led to an significant increase in cross-sectional area of pith parenchyma cells compared to wild-type plants (Fig. 6B,C). In addition, we also found that in transgenic *35S:PtrWRKY19* plants, more parenchyma cells in pith resulted in lower dry weight than the wild-type (Data not shown). Further studies will investigate whether knock-down or silencing of *PtrWRKY19* will result in an increase in biomass produce of transgenic poplar plants.

## Additional Information

**How to cite this article**: Yang, L. *et al.* PtrWRKY19, a novel WRKY transcription factor, contributes to the regulation of pith secondary wall formation in *Populus trichocarpa.* *Sci. Rep.* **6**, 18643; doi: 10.1038/srep18643 (2016).

## Supplementary Material

Supplementary Information
