# Supplementary material for: PtrWRKY19, a novel WRKY transcription factor, contributes to the regulation of pith secondary wall formation in Populus trichocarpa
Source: Sci Rep. 2016 Jan 28;6:18643. doi: 10.1038/srep18643 (PMC4730198; doi:10.1038/srep18643)
Supplement: Supplementary Information [file srep18643-s1.doc]

**PtrWRKY19, a novel WRKY transcription factor, contributes to the regulation of pith secondary wall formation in *Populus trichocarpa***

**Authors:**
Li Yang, Xin Zhao, Fan Yang, Di Fan, Yuanzhong Jiang, Keming Luo

**Supplementary information**

| **Table S1. Primer sequences used in this study.** | | | | |
| --- | --- | --- | --- | --- |
| Gene Name | Gene I.D. | Primers | Sequence | Purpose |
| AtMYB46 | At5g12870 | MBY46_F | ATCTCATCATTCGCTTTCATTC | quantitative PCR |
|  |  | MBY46_R | TGATCATGTTTCCCGTTGG | quantitative PCR |
| AtMYB63 | At1g79180 | MYB63_F | GAACAGCTCAGGCTCAAGAGCAAC | quantitative PCR |
|  |  | MYB63_R | ATGTATCATGAGCTCGTAGTTCTT | quantitative PCR |
| AtMYB58 | At1g16490 | MYB58_F | CCAGAGAACAGAGCTCTTCAAGAG | quantitative PCR |
|  |  | MYB58_R | ATGTATGAGGAGCTCGTAACTCTC | quantitative PCR |
| AtNST1 | AT2G46770 | NST1_F | GCTTAACGGACCCACATCATATTC | quantitative PCR |
|  |  | NST1_R | TTATCCACTACCATTCGACACGTG | quantitative PCR |
| AtNST2 | AT3G61910 | NST2_F | CATCAAGATCGATCTCGATG | quantitative PCR |
|  |  | NST2_R | GTCTTTCGCATCCCGATTCT | quantitative PCR |
| AtC3H14 | At1g66810 | C3H14_F | CTCTTCCTCACCACCATCTCG | quantitative PCR |
|  |  | C3H14_R | CGGTTAAGGCAAAGCTCGTA | quantitative PCR |
| AtC4H | At2g30490 | C4H_F | ATGTTCGATAGAAGATTTGAGAGTG | quantitative PCR |
|  |  | C4H_R | CAACAAAGTACTTCTTGAAAAGAGC | quantitative PCR |
| AtPAL1 | At2g37040 | PAL1_F | ACAGAGCTTTTGACCGGAGA | quantitative PCR |
|  |  | PAL1_R | CACTTCACAGACAATCATTTG | quantitative PCR |
| AtF5H2 | AT5G04330 | F5H2_F | ATCATGGATGTGATGTTCGG | quantitative PCR |
|  |  | F5H2-R | ATCTCGGTTAGCACCCATTC | quantitative PCR |
| At4CL1 | AT1G51680 | 4CL1_F | CTCCGGTGTCTGGATCAACT | quantitative PCR |
|  |  | 4CL1_R | GAAATCTGGTGCTGCTCCTC | quantitative PCR |
| AtHCT | At5g48930 | HCT_F | CTCTTTCCAAAGCCCTTGTC | quantitative PCR |
|  |  | HCT_R | TCAGCCACAACGAAGAGAAC | quantitative PCR |
| AtCCR1 | AT1G15950 | CCR1_F | TCCAGATGATCCGAAGAACA | quantitative PCR |
|  |  | CCR1_R | CGCCTTAAGAGCCTCGTAGT | quantitative PCR |
| AtCOMT | At5g54160 | COMT_F | GTCGATTGCATTATGTTGGC | quantitative PCR |
|  |  | COMT_R | AGCCTGATGCTTTGGCTAAT | quantitative PCR |
| AtIRX9 | At2g37090 | IRX9_F | TTTGCGGGACTAAACAACAT | quantitative PCR |
|  |  | IRX9_R | ATCGGAGGCTTTGTCTCTGT | quantitative PCR |
| AtCesA8 | At4g18780 | CesA8_F | CACTTCTTTGCCTCTTGTTGCTTAC | quantitative PCR |
|  |  | CesA8_R | GAAGCTCGAGGACACTCGTTAAGAT | quantitative PCR |
| AtCCoAOMT7 | At4g26220 | CCoAOMT7_F | ATGGTATCACTATTTGCAGGAGGT | quantitative PCR |
|  |  | CCoAOMT7_R | CACACAGGAACCAAAATATCACAT | quantitative PCR |
| AtCesA4 | AT5G44030 | CesA4_F | GAGTGATGATAAAACGATGAGCAG | quantitative PCR |
|  |  | CesA4_R | TCTCAAAATTCTTCTGCGACATTA | quantitative PCR |
| Atactin2 | AT3G18780 | Act2-RT-for1 | GTCCAGGAATCGTTCACAGAAAAT | quantitative PCR |
|  |  | Act2-RT-rev1 | GGGAAATGAAACAAACAAATGGAG | quantitative PCR |
| AtCAD6-F | AT4G37970 | CAD6-F | CGAGTCTCTCAAACGCAGTG | quantitative PCR |
|  |  | CAD6-R | GTTAGGTGGAGTCGGTCACA | quantitative PCR |
| AtF3H-F | At3G51240 | F3H-F | CATCAGGCCGTGGTGAACT | quantitative PCR |
|  |  | F3H-R | AAATAAACACAAAACACACCGAGC | quantitative PCR |
| AtIRX14 | AT4G36890 | IRX14-F | GATTGATCAGAGAATGCCTAATACTT | quantitative PCR |
|  |  | IRX14-R | GAATCTCATCAAACAACTCCATACTA | quantitative PCR |
| AtIRX12/LAC4 | At2g38080 | LAC4-F | CAACTAAATAGGGCGACATATCAAC | quantitative PCR |
|  |  | LAC4-R | TTATTCAAACTTTTGACATGAGCA | quantitative PCR |
| AtGUT2/IRX10 | At1g27440 | GUT2-F | GTGAGAAGGCACTGAACTGGACT | quantitative PCR |
|  |  | GUT2-R | GACTTCTAATGTTTTTGAAGTGCT | quantitative PCR |
| ptrWRKY95 | Potri.014G050000 | Potri.014G050000.1-F2 | TCACTCTCCATGTGATGATTCC | quantitative PCR |
|  |  | Potri.014G050000.1-R2 | GTCAGAAATGGCTGATCTTGGG | quantitative PCR |
| PtrCAD1 | EU603306.1 | PtrCAD1-F | AAGTTTGTGGTGAGAATTCCTGATG | quantitative PCR |
|  |  | PtrCAD1-R | AAACTGTCAATCCAGCGCACAATAG | quantitative PCR |
| PtrCCR2 | EU603310.1 | PtrCCR2-F | GGGCTGGTGGTTTCATTGCTTCTT | quantitative PCR |
|  |  | PtrCCR2-R | TTCTTGGGATCAGCTGGGTTCCT | quantitative PCR |
| PtrC4H2 | EU603302.1 | PtrC4H2-F | GAAATGTGCAATTGATCATATTTTG | quantitative PCR |
|  |  | PtrC4H2-R | ATTGCAGCAACATTGATGTTCTCC | quantitative PCR |
| PtrSND1-A1 | Potri.011G153300 | SND1-A1-rF | TAGGCTTGATGACAGCACCCATGAA | quantitative PCR |
|  |  | SND1-A1-rR | TCTAAATACCCGGCAAACCACCCAA | quantitative PCR |
| Ptr18S | AY652861 | Ptr18S-F | CGAAGACGATCAGATACCGTCCTA | quantitative PCR |
|  |  | Ptr18S-R | TTTCTCATAAGGTGCTGGCGGAGT | quantitative PCR |
| PtrWRKY95 | Potri.014G050000 | Potri.014G050000.1-F1 | CAATTCATGGTGGAGGAGCT | semi-quantitative PCR |
|  |  | Potri.014G050000.1-R1 | GTCAGAAATGGCTGATCTTGGG | semi-quantitative PCR |
| AtWRKY12 | AT2G44745 | WRKY12-F | AGGAAGAGAGACAATCATGG | semi-quantitative PCR |
|  |  | WRKY12-R | TCGTCAAGAACAGCCTTCAC | semi-quantitative PCR |
| AtUBC | AT5G25760.1 | UBC-F | GCAACCTCCTCAAGTTCGATTC | semi-quantitative PCR |
|  |  | UBC-R | GGCGTGTATACATTTGTGCCAT | semi-quantitative PCR |

**
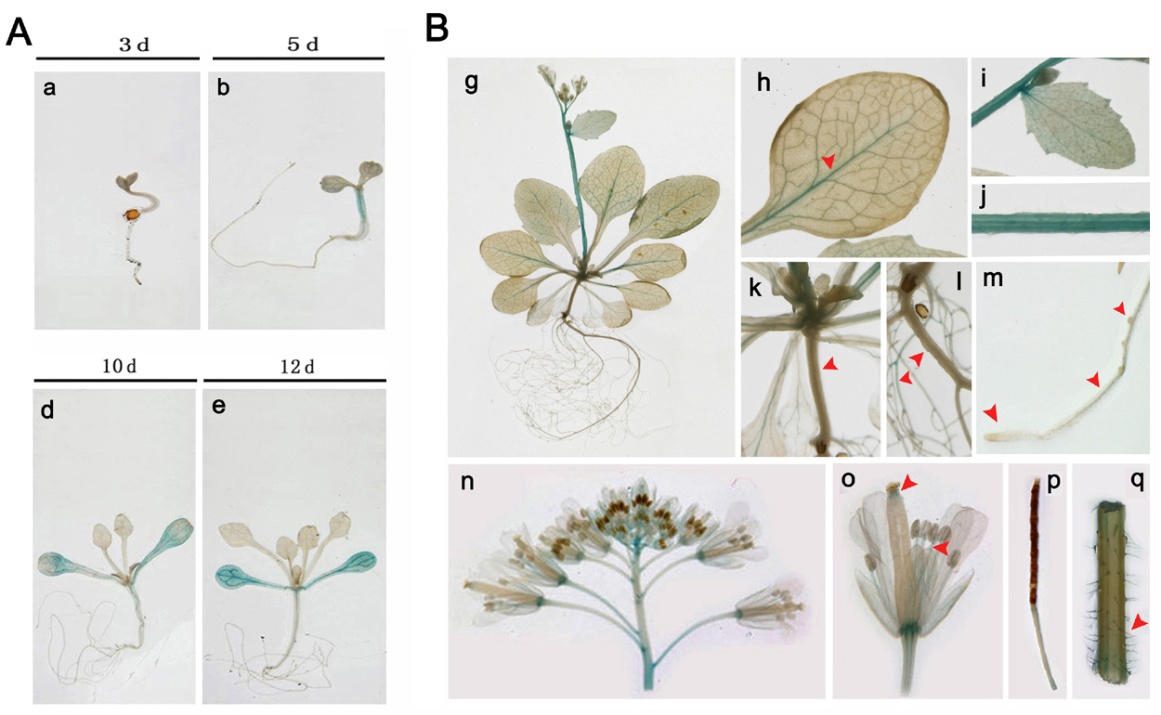
**

**Figure S1. Expression pattern of the *PtrWRKY19* Promoter.**

The 1463 bp promoter of the *PtrWRKY19* gene was inserted into the upstream of the GUS gene in the vector pCXGUS-P and the resulting vector was transformed into *Arabidopsis* plants. GUS activity was examined during different development stages of transgenic plants.(A) Whole seedling. (a) 3-day-old , (b) 5-day-old, (c) 10-day-old, (d)12-day-old -15d. (B) GUS staining of flowering plants. (g) 30-day-old seedling. GUS activity was detected in the vein (h), petiole (i)and young leaves (j) of transgenic plants. (k) No GUS staining was found in elongation hypocotyls and roots of transgenic plant.(m) The lateral roots showed a slight GUS staining.(n) GUS activity was found in the filament (n) and adhesion zones of stigma (o) but no GUS activity in mature silique (p).GUS activity was also detected in developing inflorescences.


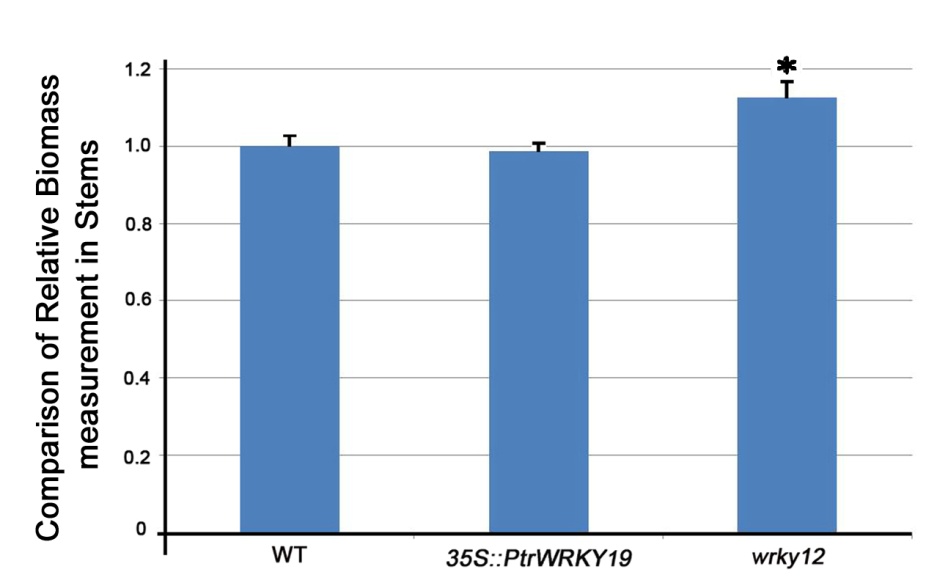


**Figure S2. Total biomass measurement of the stems from WT, *35S-PtrWRKY19* and *wrky12* mutant** **complemented with *35S-PtrWRKY19*.**

The biomass of the wild type plants was set to 1. The data correspond to the mean ±SD (n=3) from triplicate analyses (Student’s *t* test; *P<0.05). No significant decrease in the biomass density relative to the *35S-PtrWRKY19* Arabidopsis plants.

**
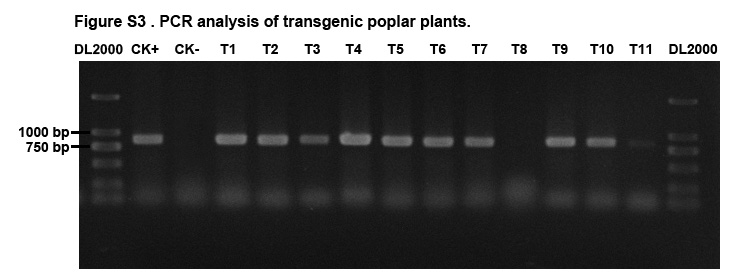
**

**Figure S3. PCR analysis using gene-specific primers was employed to confirm the presence of the transgenes in transformed plants.**

An expected amplification product for the *HPT* gene was obtained from all transgenic lines tested except T8 line, whereas no signal was detected from untransformed plants.CK+, plasmid DNA; CK-, water.

**
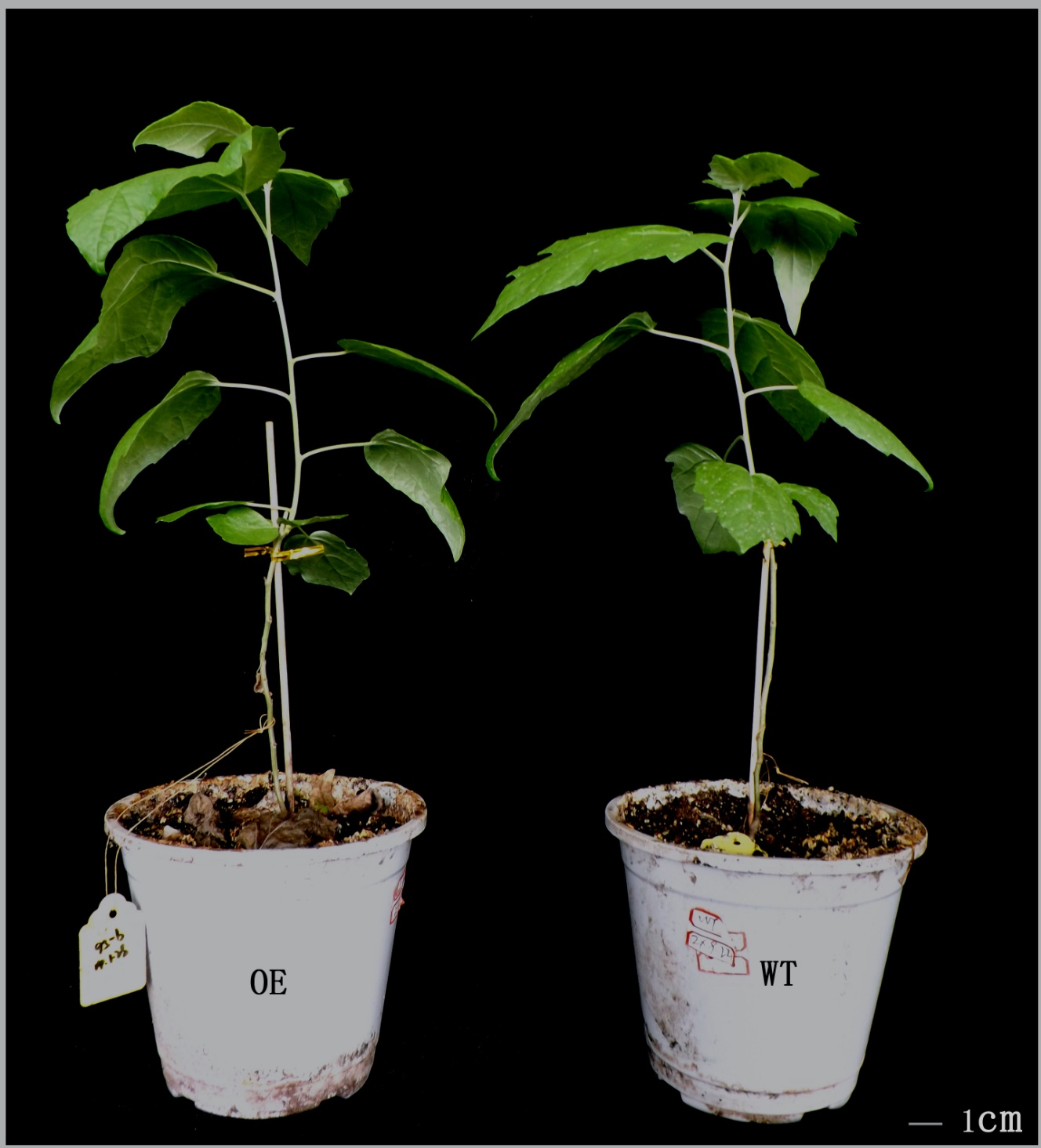
**

**Figure S4. Phenotypes of the wild-type (Right) and transgenic *35S:PtrWRKY19* poplar plants (left).**


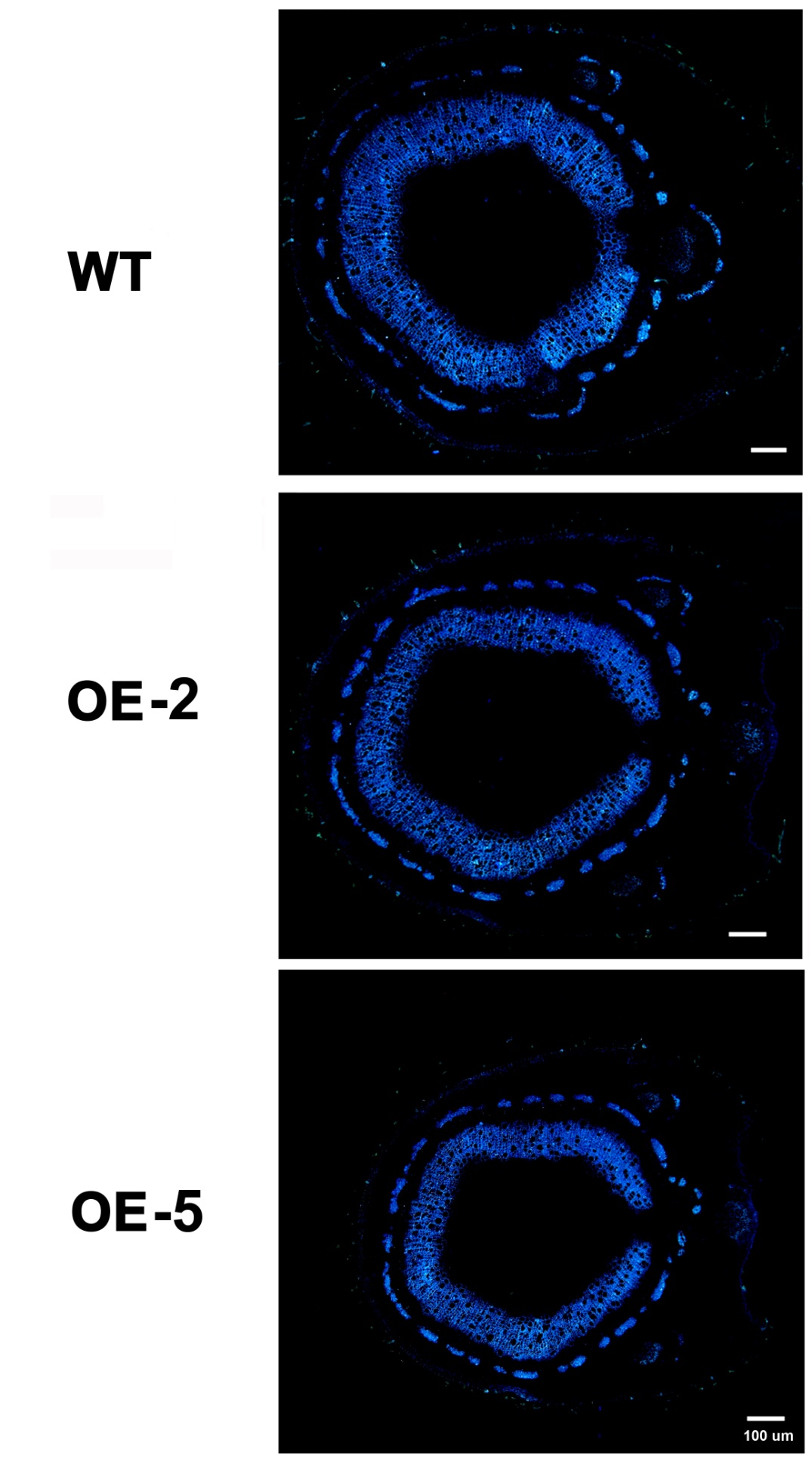


**Figure S5. UV autofluorescence of stem cross-sections of the wild-type and transgenic 35S:*PtrWRKY19*lines.**

>Potri.019G130700

TAAGGAGATGAAAAGCATGCAATTTATATTTAGAAAAGGCGAGAAGAAGAAGAGATAAGGAAGAAAAATAAATGTTTAAGGGAAAGCGAAAGAAATCCTTTACGATTTAGGTGGGGGTAGATGGGCATGCAGGAGTTGAAACTGAAGGCCGCACATACGCCGTTCAAAGTAACGGTAAAGTCTAGTCGGCTGTTTAATGGTTGACTTTTCTTTCTTTTCAGTTATTTCAATTGAGGATTGATTTCTTCCAAATTTCGACTTTTTCCTCAAAACATGTTTAACATTTGCAATTTGCACCTCCGCAAAACGTTGATGACATTCACAGCATAGCATGACACCAGTCCTTTGAACTAGGATGATAAGCTTGTTCCTTAATTAATTTATTCAAACTAGTTAGATTTTGTAGCATTGAAAATAAATAAATTATTTTCTTAATTAATCGGTATTCCTCAATTTAATAATTTATTTTATTAAAAAAACCCATTTCGCCGTTAACCTTTTTTTATTTACTCTATTCTCTTAATAAAACAAAATACAAAATGAAATGAAAAACGGGAAAGTATTTTTAAATGCTAAATGTATATATACCCAATAAAAATGTAAGTTACCAAGTATATCTAAAATAATAAATTAAATTAATTAATTTAATCATAAACATTAACCCATTAATTGTATTGATATTAGCCATTTAAATTTTATACACTACAATTAAAATTTAATTGGCTATATCAATAAAATTTATTAATTATAAAAACACCTATAATTAATTATAATTTGAGATCAATATTCTAACACGAGGGACTATTGACGAAGTCCCGATTAATGAATATTCTTGAAGTGTGTGTATATATACATATATGTATGTGTAAAACCTGTTGGCATAAAATAAAACCCGAGTTATGTATGAAGAGGAATTCTTTGATTTTTGTTAAAGGATCAAAATAATATTGATTTGAAAAAAAATTAAAAAGAATAGTGAATGGAATAAGTGGAGATCATTTTGGATTCTTGAATGTGTTAGTTAGTTTTTCTATTTTTTTAACTTAAATCAAGGTTTTAAATTAATTTTAAAAAATATAATTTAATTGGTCAGATTCTGAATTCAGTTTTTAAAGATCATCAATTTAAATGCTATAAAATTTAAGATTATTAAAAATTTACATAAAAATTAACTTTAAAATTTTAAAAAATTAATCAAGAAATATAAAAATTAACTCAGAAACTACGGTAAATAAAAATAAAAATAATAAGGTCTTGTATTGAGATGAAGAGAGTCGCGCTTGTATGCGTATATATATAAGGAATTTGTGACATCATGTGCCTCACCTACTACCTCTACAACGATCAATGCTGTGTTGTCCCTCTCCTATCACACCTCAGAACTAACGCCGTTAACTCTGTTTCAGAAACCTAACTGGAAGCCACACGTCCACCTAACTCATGAACTATCTTTATCTCCAACCTTCCAGTTGCTCTTTAATTCTTCTACTGCTTATAAAACCCCCTCCCAACCCCCACTCACTCCCCACCAACCACCTAGTCTCTCCTCCTCTTAATTATATTTTCTTCAATCTACCACTCCTTCTTCTCCCCAGAAATCTCTTTCAGTACTCCTTTGGATAAAGAAGATCATATTCCAGTATAATG

W-box: T/CTGACT/C

200 GTTGACT (+)

201 TTGACTT(+)

555 TCTGACC(-)

556 CTGACCA(-)

**Figure S6. The promoter sequence of *PtoC4H2*.**

At least 4 W box (TTGACC/T) elements were found in the promoter region of ***PtoC4H2***.
